# The Association between the *LPA* Gene Polymorphism and Coronary Artery Disease in Chinese Han Population

**DOI:** 10.1155/2014/370670

**Published:** 2014-03-25

**Authors:** Zi-Kai Song, Hai-Di Wu, Hong-Yan Cao, Ling Qin

**Affiliations:** Department of Cardiology, The First Hospital of Jilin University, 3302 JiLin Street, Changchun 130031, China

## Abstract

Lp(a) has been well known as an independent risk factor for coronary artery disease (CAD). The *LPA* gene, as it encodes apo(a) of the Lp(a) lipoprotein particle, was associated with increased risk of CAD. The purpose of this study was to analyze the relationship between the polymorphisms of *LPA* gene and CAD in Chinese Han population. Five SNPs (rs1367211, rs3127596, rs6415085, rs9347438, and rs9364559) in the *LPA* gene were genotyped using Sequenom MassARRAY time-of-flight mass spectrometer (TOF) in 560 CAD patients as case group and 531 non-CAD subjects as control group. The numbers of these two groups were from Chinese Han ancestry. The results showed that allele (*P* = 0.046) and genotype (*P* = 0.026) of rs9364559 in the *LPA* gene was associated with CAD. The frequency of rs9364559 minor allele (G) in case group was obviously higher than that in control group. Results of haplotype analysis showed that 4 haplotypes which contained rs9364559-G were associated with increased risk of CAD in this population. This study explored rs9364559 in the *LPA* gene may be associated with the pathogenesis of CAD; and the risk of CAD might be higher in the population carrying 4 haplotypes of different blocks in the *LPA* gene.

## 1. Introduction

Coronary artery disease (CAD) has become a major cause of death and disability, accounting for up to 40% of all lethal events [1]. However, the pathogenesis of CAD is not fully understood yet. Many studies showed that CAD is mainly caused by genetic and environmental factors [2]. Many genomewide association studies (GWAS) have identified several novel susceptibility gene loci for CAD [3–6]. Variants of lipid metabolism-related genes have received the widespread attention of scientists.

Elevated Lp(a) levels has been considered to be an independent risk factor for CAD [7–10]. In 1963, Berg first discovered the lipoprotein (a) (Lp(a)) in the separation of plasma lipoprotein [11]. Lp(a) is produced in the liver [12] and circulates in the plasma, which is an LDL-like particle that consists of an apolipoprotein (a) (apo(a)) moiety linked to one molecule of apolipoprotein B100 via a disulfide bond [13]. The study by Boerwinkle et al. shown that the plasma Lp(a) concentration was mainly affected by apo(a) gene polymorphism, which accounts for 91% of the variation [14].

A genome wide association study showed that a cluster of genes-solute carrier family 22 member 3 (*SLC22A3*), lipoprotein (a)-like 2 (*LPAL2*), and lipoprotein (a) (*LPA*) on chromosome 6q26-27 was strongly associated with CAD risk [15].* LPA* gene in the 6q26-27 region encodes apo(a) of the Lp(a) lipoprotein particle [9]. Several* LPA* gene polymorphisms have been identified that have significant associations with an elevated Lp(a) level and a reduced copy number of K4 repeats [16]. However, the relationship between* LPA* gene polymorphisms and CAD varies across races/ethnicities as there are large differences between minor allele frequencies. In the study by Clarke et al. rs3798220 and rs10455872 are strongly associated with serum Lp(a) levels in Caucasians [9]. However, the variant allele frequency (G allele) of rs10455872 is less than 1% in Chinese people without CAD. Another* LPA* SNP rs6415084, within the same haplotype block as the KIV-2 variation, was significantly associated with both Lp(a) concentration and KIV-2 copy number in the same direction in all 3 ethnicities. Therefore, the objective of our study was to investigate a possible association between* LPA* gene polymorphisms and the risk of CAD in a case-control study of the Chinese Han population.

## 2. Materials and Methods

### 2.1. Study Subjects

All subjects included 560 CAD patients (308 males and 252 females) and 531 controls (270 males and 261 females) in order to undertake a genetic analysis for association between the* LPA* gene polymorphisms and CAD. All the subjects including the case group and the control group used for this study were Chinese of Han descent. The case group was collected from the hospitalized patients with CAD in the cardiology department of the First Hospital of Jilin University from June 2009 to September 2012.

Diagnose was carried independently by at least two well-trained physicians based on the following criteria. All patients were identified with CAD by coronary computed tomographic angiography (SIEMNS Somatom Definition AS+128 row spiral CT). CAD was defined by ≥50% stenosis in any major coronary artery. All recruited patients had evidence of CAD documented by unstable angina or myocardial infarction. Unstable angina and myocardial infarction were confirmed by Chinese guidelines (Chinese Medical Cardiology Subcommittee, Chinese Editorial Committee of Cardiology Journal, 2010; Chinese Medical Cardiology Subcommittee, Chinese Editorial Committee of Cardiology Journal, 2007). Patients with nonatherosclerotic vascular diseases, congenital heart disease, cardiomyopathy, valvular disease, renal or hepatic disease, and cancer were excluded. All control subjects had ECG, chest X-ray, and serum analysis. They were classified as healthy subjects based on their normal physical examination results coupled with the absence of personal or family history and reasons for being suspected CAD.

The presence of cardiovascular risk factors, including diabetes mellitus (fasting blood glucose ≥7.0 mmol/L and/or using glucose-lowering medication, including insulin), blood pressure, and cigarette smoking, were obtained from all participants. Hypertension was defined according to seated blood pressure readings of 140/90 mmHg and higher and/or subjects' receiving antihypertensive medication. In this study, hypercholesterolemia was defined as a serum total cholesterol level of 200 mg/dL or more, and a smoking habit was defined as a daily intake of >10 cigarettes [17].

All the subjects have written informed consent for the study, which was approved by ethics committee of Jilin University, Changchun, China.

### 2.2. Laboratory Examination

Before starting the study, all participants underwent an initial screening assessment that included medical history, vital signs, a 12-lead electrocardiogram, and measurement of lipid variables and novel risk factors. Venous blood was collected in the morning after an overnight (8–12 hours) fast. Serum/plasma samples were frozen and stored at −80°C prior to analysis. All measurements were performed in a central laboratory.

### 2.3. SNP Selection, Identification, and Genotyping

Tagging SNPs were chosen from genotyped SNPs in Chinese Han population (CHB) of the HapMap project (Phase I database). The candidate SNPs were restricted to minor allele frequency bigger than 15% in HAPMAP-CHB database (http://www.hapmap.org/). Genomic DNA used for PCR amplification was extracted from the whole blood sample using a DNA extraction kit (Takara, China). Primers of amplification and extension were used AssayDesigner3.1 software. Amplification and extension primers sequences of five loci in* LPA* gene were in Table 1. Genotypes were assigned real time using Typer 4.0 software (Sequenom). As quality controls, 5%–10% of the samples were genotyped in duplicate. No inconsistencies were observed. Controls distributed in each 384 well plates were also consistent. Cluster plots were made of the signals from the low and the high mass allele.

### 2.4. Statistical Analysis

Data were expressed as percentages of total for categorical variables or mean ± SD. The statistical analyses on the characteristics of the subjects were performed with Pearson *χ* ^2^ test for the categorical variables such as sex, smokers, drinkers, hypertension, and diabetes and with Student's *t*-test for the continuous variable of age, TC, and TG with normal distribution. SPSS 16.0 was used for the above analyses.

The Hardy-Weinberg equilibrium for the genotypic distributions of SNPs was tested by the chi-square (*χ* ^2^) goodness-of-fit test. The Haploview program (version 4.1) was applied to estimate the linkage disequilibrium (LD) measures (*D*′ and *r* ^2^) between paired SNPs. Allelic, genotypic, and haplotype analyses were performed with SHEsis software. Software website is http://analysis.bio-x.cn/. Results are expressed as odds ratio (OR) and 95% confidence intervals (CI). *P* < 0.05 was used as the criterion of statistical significance, and all statistical tests were two sided.

## 3. Results

### 3.1. Characteristics of Participants

In Table 2, the demographic and clinical characteristics of the 560 CAD patients and 531 control subjects have been listed. There was no significant difference of mean ages, sex, BMI, and serum TG level between case and control groups. However, compared with control group, CAD group had more smokers and more individuals with hypertension and diabetes. Additionally, compared with control group, CAD group had higher level of serum TC.

### 3.2. Allele and Genotype Analysis

The distributions of alleles and genotypes of five loci among participants were presented in Tables 3 and 4, respectively. Analysis with the SHEsis software showed that genotype and allele frequencies of rs9364559 were significantly higher in case group than that in control group, respectively (*χ* ^2^ = 7.302, *P* = 0.026; *χ* ^2^ = 3.981, *P* = 0.046). However, for another four SNPs, there were no differences in genotype and allele frequencies between two groups.

### 3.3. Linkage Disequilibrium (LD) Analysis

Rs1367211 and rs6415085, rs1367211 and rs9347438, and rs3127596 and rs9364559 are all located in different LD block on 6q26-27 region (*D*′ = 1.000, *r* ^2^ = 0.085; *D*′ = 1.000, *r* ^2^ = 0.149; *D*′ = 0.999, *r* ^2^ = 0.082).

### 3.4. Haplotype Association Analysis

All haplotypes with a frequency above 3% were included in the following analysis. In the haplotype association analysis, one haplotype was treated as a single variant, and all the other haplotypes were collapsed into the alternative allele to test its association with CAD. Four haplotypes formed by different blocks were associated with increased risk of CAD in this population (Table 5). GATG (which was made up by rs1367211, rs3127596, rs6415085, and rs9364559), ATG (which was made up by rs3127596, rs9347438, and rs9364559), GTG (which was made up by rs1367211, rs9347438, and rs9364559), and TG (which was made up by rs6415085 and rs9364559) reached the single-point significance level (*P* = 0.034, 0.036, 0.049, and 0.039).

## 4. Discussion

Based on our results, we found the association between rs9364559 in the* LPA* gene and risk of CAD in Chinese Han population; haplotypes GATG (which was made up by rs1367211, rs3127596, rs6415085, and rs9364559), ATG (which was made up by rs3127596, rs9347438, and rs9364559), GTG (which was made up by rs1367211, rs9347438, and rs9364559), and TG (which was made up by rs6415085 and rs9364559) were risk haplotypes for CAD in Chinese Han population. Therefore, rs9364559 in the* LPA* gene has played a significant role in the pathogenesis of CAD in Chinese Han population. This result agrees with the previous results in other populations [9, 18]. However, the result that rs9364559 was associated with CAD in Chinese Han population is reported for the first time.

High level of Lp(a) in plasma has been confirmed to be associated with an increased cardiovascular risk [9, 19–23], which is predominantly determined by apo(a) gene [14]. Apo(a) size polymorphism and nonsize polymorphism in the* LPA* gene all affect Lp(a) level [24, 25]. Rs3798220, located in the protease-like domain of apo(a), and rs10455872, which maps to intron 25, have repeatedly been associated with an increased Lp(a) level and a reduced copy number of K4 repeats in Caucasians [9]. These variants were both associated with plasma Lp(a) levels, and the association between these gene variants and CHD was abolished when plasma Lp(a) levels were entered into the model [9]. However, the study by Lamon-Fava et al. showed that rs3798220 was significantly associated with Lp(a) levels, and it was not a significant predictor of CHD [26]. And the association between apo(a) gene polymorphism and CAD varies among races. Both of these SNPs had no significant effect on serum Lp(a) levels in Chinese Han population [27]. Our study found that genotype and allele frequencies of rs9364559 in the* LPA* gene were significantly higher in case group than that in control group, respectively. Therefore, rs9364559 might affect risk of CAD. In this study, 4 haplotypes formed by different blocks in the* LPA* gene were risk haplotypes for CAD in Chinese Han population, and these haplotypes all contain rs9364559-G. Consequently, the above results further proved that* LPA* gene is associated with CAD, which is consistent with previous studies.

However, in haplotype association analysis, there is no difference for haplotype frequencies formed by five loci in the two groups. Less number of samples may be the main cause of the problem. Therefore, further research conducted in a lager sample size in different race is necessary.

In conclusion, our case-control study explored the association between five SNPs (rs1367211, rs3127596, rs9347438, rs6415085, and rs9364559) in the* LPA* gene and CAD in Chinese Han population for the first time. Rs9364559 in the* LPA* gene may be associated with risk of CAD in Chinese Han population, and with 4 haplotypes population formed by different blocks in the* LPA* gene may be associated with increased risk of CAD in Chinese Han population. Therefore,* LPA* gene is strongly associated with CAD in the Chinese Han population, which agrees with previous study.

## Figures and Tables

**Table 1:** Amplification and extension primers sequences of five loci in *LPA* gene.

SNPs	Forward primer (5′-3′)	Reverse primer (5′-3′)	Extension primer (5′-3′)
rs1367211	ACGTTGGATGGTGGATTAGGTTCAGAATGC	ACGTTGGATGGCTCTATGCTGGAAAACTGG	ATGGGAAACTGGATTATTGAACAGGCAC
rs3127596	ACGTTGGATGTATGGGATGCCATCCTTCTC	ACGTTGGATGAAGCACTGCAGATGCTTGAG	AGTAATATGCTCATAAGTTCCC
rs9364559	ACGTTGGATGTTTTGTCCATGTACCTGCCC	ACGTTGGATGAGGAGAGGAAGAGCAAAAGC	ATGAGAATTAGGAAGTAAACAGAC
rs6415085	ACGTTGGATGACTTTAGCATATGTAGAGG	ACGTTGGATGCACTTCCAATTATTCCCCAC	TTATTCCCCACATGATTTAGG
rs9347438	ACGTTGGATGGTTCTAGCCTTTGGGTGTAG	ACGTTGGATGTTCAGCCCATGGAAACTAGG	CCATGGAAACTAGGATGTAGA

**Table 2:** Base characteristics of case group and control group.

Variable	Case group (*n* = 560)	Control group (*n* = 531)	*P* value
Age (year)	62.39 ± 10.90	61.82 ± 12.77	0.447
Sex (%)	55.0	50.8	0.170
Smoking (%)	38.3	23.3	0.000
Drinking (%)	22.3	22.7	0.903
Hypertension (%)	56.1	30.7	0.000
Diabetes mellitus (%)	24.0	12.1	0.000
TC (mmol/L)	5.22 ± 4.28	4.62 ± 1.24	0.002
TG (mmol/L)	1.76 ± 1.22	1.74 ± 1.20	0.962
BMI (kg/m^2^)	24.19 ± 2.68	24.01 ± 3.31	0.431

**Table 3:** SNPs loci allelic frequency distribution of *LPA* gene and the relationship with coronary heart disease.

SNPs	Controls		Cases		*χ* ^2^	*P*
	A	G	A	G		
rs1367211	204	842	216	860	0.109	0.741
rs3127596	889	157	914	170	0.186	0.667
rs9364559	738	306	716	358	3.981	0.046

	G	T	G	T		

rs6415085	769	257	796	280	0.262	0.609

	C	T	C	T		

rs9347438	395	653	407	671	0.001	0.976

**Table 4:** SNPs loci genotype frequency distribution of *LPA* gene and the relationship with coronary heart disease.

SNPs	Controls			Cases			*χ* ^2^	*P*
	AA	GA	GG	AA	GA	GG		
rs1367211	23	158	342	20	176	342	0.967	0.616
rs3127596	379	131	13	387	140	15	0.186	0.911
rs9364559	266	206	50	231	254	52	7.302	0.026

	GG	GT	TT	GG	GT	TT		

rs6415085	346	77	90	352	92	94	1.574	0.455

	CC	TC	TT	CC	TC	TT		

rs9347438	75	245	204	77	253	209	0.004	0.998

**Table 5:** Haplotype analysis of all blocks between two groups.

Haplotype	case	control	*χ* ^2^	*P*	OR	95% CI
rs1367211, rs3127596, rs6415085, rs9364559						
GATG	72.95 (0.069)	47.80 (0.047)	4.486	0.034	1.497	1.028–2.179

rs3127596, rs9347438, rs9364559						
ATG	176.52 (0.166)	138.94 (0.133)	4.384	0.036	1.293	1.016–1.645

rs1367211, rs9347438, rs9364559						
GTG	42.38 (0.040)	25.66 (0.025)	3.890	0.049	1.643	0.998–2.703

rs6415085, rs9364559						
TG	75.08 (0.071)	50.13 (0.049)	4.278	0.039	1.472	1.019–2.127

Frequency <0.03 in both control and casegroups has been dropped.
